# Long-term skeletal and dentoalveolar effects of hybrid rapid maxillary expansion and facemask treatment in growing skeletal Class III patients: a retrospective follow-up study

**DOI:** 10.1186/s40510-022-00429-0

**Published:** 2022-09-30

**Authors:** Giuliano B. Maino, Francesca Cremonini, Giovanna Maino, Emanuele Paoletto, Matteo De Maio, Giorgio Alfredo Spedicato, Mario Palone, Luca Lombardo

**Affiliations:** 1 Postgraduate School of Orthodontics, University of Ferrara, Via Luigi Borsari 46, 44121 Ferrara, Italy; 2 Lab Orthomodul, Via S. Vincenzo, 61, 36016 Thiene, VI Italy; 3 School of Dentistry, University of Ferrara, Ferrara, Italy; 4 Bicocca University, Piazza dell’Ateneo Nuovo 1, 20126 Milan, MI Italy

**Keywords:** Skeletal expansion, Miniscrews, Palatal anchorage, Class III malocclusion, Facemask

## Abstract

**Background:**

Regardless of the treatment protocol, stability in Class III patients always represents a major concern. The aim of this study was to assess the short and long-term skeletal and dentoalveolar modifications in a group of class III patients treated with hybrid rapid maxillary expander (RME) and facemask (FM). Indeed, no long-term studies have been conducted yet with the objective of evaluating the effects of this kind of approach when applied to patients who have already gone thought their peak of growth.

**Material and methods:**

27 patients with skeletal Class III malocclusion were treated using hybrid RME according to alternating rapid maxillary expansion and constriction (ALT-RAMEC) protocol, followed by 4 months of facemask therapy. After the orthopaedic phase, each patient underwent orthodontic treatment with fixed multibracket appliances. A mean follow-up of 7 years, 10 months was performed. Pre-treatment (TO), post-treatment (T1) and follow up (T2) cephalometric tracing were analysed, comparing dental and skeletal measurements.

**Results:**

Point A advanced by a mean of 3.5 mm with respect to VerT, then relapsed by 0.7 in the post-facemask period, thereby yielding of a mean advancement of 2.7 at T2. The sagittal relationship significantly changed after RME + facemask protraction (3.8° of ANB and 5.189 mm of Wits). Although both Wits and ANB values worsened over time, the improvement from T0 is still appreciable at T2.

**Conclusion:**

Despite the physiological relapse due to mandibular growth, the long-term cephalometric follow-up confirms the maintenance of all positive outcomes of the previous orthopaedic treatment with hybrid RME and facemask.

## Introduction

### Background

Skeletal Class III malocclusion is one of the most challenging orthodontic corrections to perform. The resolution of this type of malocclusion usually requires effective and early intervention. Indeed, timing is crucial for providing a more favourable growth pattern and improving the occlusal relationship [1]. In the field of Class III interceptive treatment, there is moderate evidence to show that the use of facemask results in positive improvements in both skeletal and dental development in the short term [2]. However, there is a lack of evidence for the long-term benefits [3]. Patients subjected to early facemask therapy have an anterior crossbite relapse rate of around 25% [4], associated with late residual mandibular growth and pronounced intermaxillary discrepancies [5].

Sagittal correction must always be preceded by normalization of the upper transverse diameters, which appear reduced in the presence of hypoplastic maxilla in skeletal Class III malocclusion [6].

A systematic review evaluating the effect of RME treatment on sutures in all three dimensions pointed out that particularly the zygomaticomaxillary and frontomaxillary sutures are affected by the maxillary expansion. For this reason, the skeletal expansion has some important clinical implications and may explain the forward and downward displacement of the maxillary, which can be beneficial in Class III corrections in young patients [7].

Some other immediate perceived benefits associated with rapid maxillary expansion (RME) in conjunction with maxillary protraction therapy include disarticulation of the circummaxillary sutures to determine more pronounced orthopaedic effects [8]. Even though RME has been recommended in Class III correction, the circummaxillary sutures were found to be less disarticulated with the use of RME as compared to alternating rapid maxillary expansion and constriction (ALT-RAMEC) [9], which was first introduced by Liou [10]. Weaking and opening the circumaxillary sutures by alternating expansion and compression of the maxillary complex are able to enhance Class III mechanics [11].

Taking into consideration the ossification age of the sutures, the effectiveness of combined palatal expansion and facemask maxillary protraction is maximal when patients are younger than 10 years old. After that age, undesirable side effects, which include excessive forward movement and extrusion of the upper molars, excessive proclination of upper incisors, and increased lower face height, can easily result from the traditional approach [12].

For patients who are close to the end of craniofacial growth, Maino et al. [13] developed a 3-dimensional surgical guide to provide safe palatal miniscrews insertion (MAPA). A hybrid RME anchored to both the bone and the teeth was developed, used with ALT-RAMEC protocol and consequently named SKAR III (Skeletal Alt-RAMEC for Class III). After disarticulation of the circummaxillary sutures, 4 months of facemask therapy is foreseen [14].

Regardless of the treatment protocol used, stability in growing Class III patients always represents a major concern. Traditional RME/FM treatment was shown to be an effective solution for correcting skeletal class III malocclusion in the long term, with favourable skeletal effects when used before the pubertal growth spurt [8]. Previously, the Alt-RAMEC/FM approach has also been evaluated when applied to very young patients. By comparing lateral cephalograms taken before, after treatment and at post-pubertal observations, the protocol cannot be recommended as the first choice in very young subjects compared to the conventional RME/FM protocol [15].

On the other hand, no long-term studies have been conducted yet with the objective of evaluating the effects of Skar III applied to patients who have already gone thought their peak of growth.

### Objectives

The aim of this study was to assess both the short- and long-term skeletal and dentoalveolar modifications in a group of patients treated using the Skar III appliance with the protocol previously described.

## Material and methods

### Study design

This retrospective study was performed in accordance with the 1975 Declaration of Helsinki ethical standards and its later amendments, and comparable ethical standards. The study design was approved by the Ethics Committee of the Ferrara University Postgraduate School of Orthodontics (Via Luigi Borsari 46, Ferrara, Italy; approval number 3/2021).

### Setting and participants

The study group consisted of 27 patients (15 girls and 12 males; mean age 11 years, 4 months ± 22 months), treated using the same combined hybrid RME and facemask protocol by two operators (G.B.M., L.L.) in their private practice, in a recruitment period of 2 years. Inclusion criteria for the study were (1) Caucasian patients; (2) Patients with noncleft, no syndromic Class III malocclusion consecutively treated with the Alt-RAMEC and maxillary protraction technique; (3) Late deciduous or permanent dentition at the beginning of treatment; (4) no mandibular asymmetries; (5) Skeletal class III malocclusion with no functional shift; (5) Sufficient cooperation of the patients during treatment; (6) Long follow-up records available. The following exclusion criteria were applied: craniofacial syndromes and previous orthopaedic or orthodontic treatment.

### Clinical intervention

**Fig. 1:**
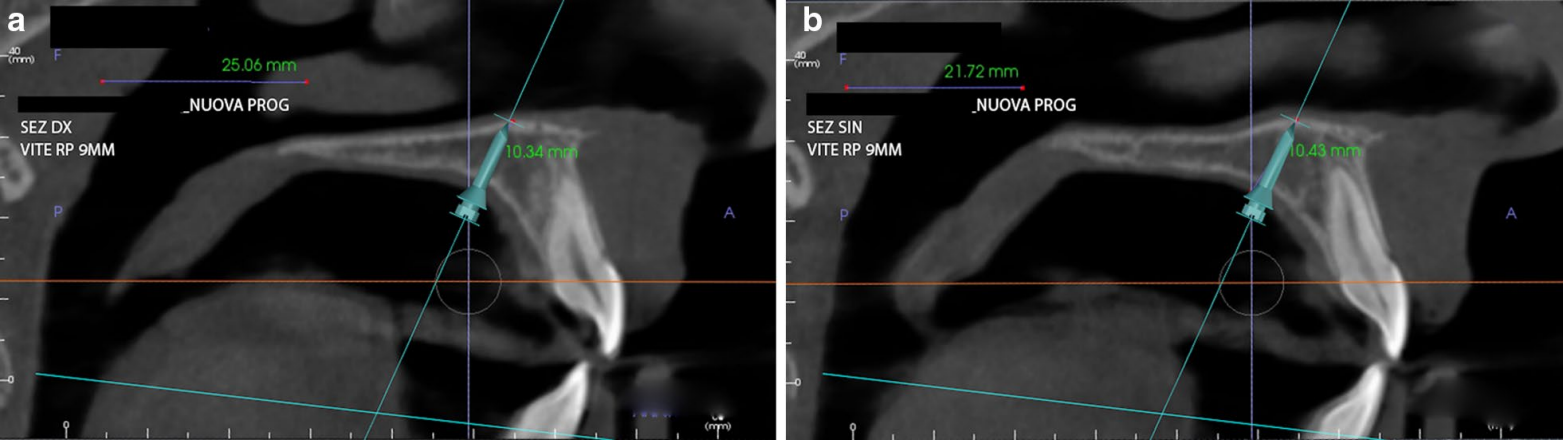
**a**, **b** Planning of miniscrews insertion on CBCT

**Fig. 2:**
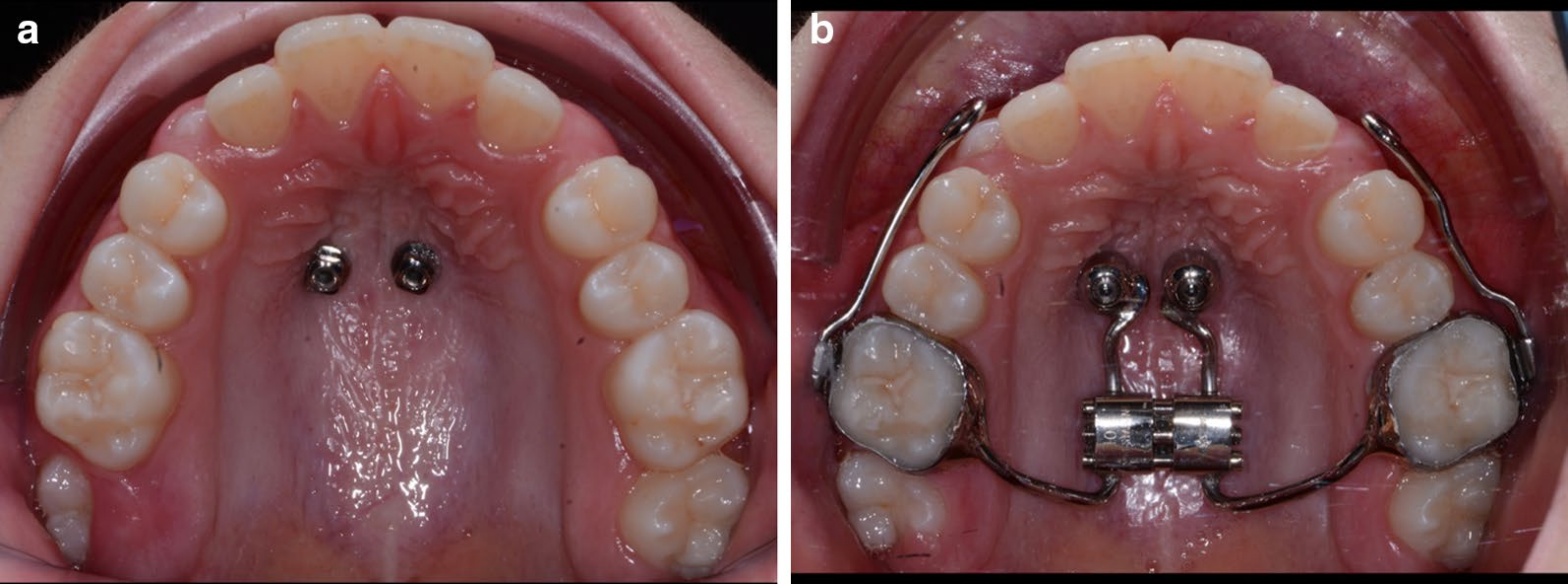
**a**, **b** SKAR III orthodontic device

The optimal sites and direction of miniscrews insertion were identified on a cone-beam computed tomography (CBCT) scan (Fig. 1a, b). After intra-oral scanning, a digital model of the maxillary arch was superimposed onto the CBCT scan, using eXam Vision (KaVo, Biberarch, Germany) and InVivo software (Anatomage, Inc, Santa Clara, CA) to identify the most appropriate anteroposterior placement sites. Then, a virtual surgical guide was designed using the same digital software [13, 14]. After miniscrew insertion (Spider Screw Regular Plus; HDC, Vicenza, Italy), the expansion device chosen in all cases was SKAR III, characterized by mixed dental and skeletal anchorage and vestibular arms for facemask (Fig. 2a, b). As in previous works [14], the Liou protocol [15], consisting of an alternating 4 activations a day in expansion for 1 week and 4 activations a day in constriction for 1 week, was applied in order to achieve maxillary expansion and suture mobilization. After five weeks, a facemask was attached near the maxillary canines using protraction elastics (400 g per side) with a downward and forward pull of 30° with respect to the occlusal plane. The prescription was 14 h per day for 4 months. After a mean time of 3 years from the end of the first orthopaedic phase, each patient underwent a non-extractive orthodontic treatment with fixed multibracket appliances to achieve the six ideal keys of occlusion.

### Data measurements

Cephalometric tracings were generated for each patient at three different time-points: T0 (pre-treatment), T1 (post-facemask treatment, 8 months after T0) and T2 (7 years, 10 months after the end of the previous protocol, patients’ mean age: 19 years, 10 months ± 21 months).

Cephalometric analysis was performed according to the method of Baccetti et al. [16]. and DeClerck et al. [17] Specifically, the stable basicranial line, drawn tangent to the lamina cribrosa of the ethmoid bone and through the most superior point of the anterior wall of the Sella turcica at the junction with the tuberculum sellae (Point T) [18], and then the vertical T (VertT), perpendicular to the basicranial line passing through point T, were traced. Neither of these lines change over time after the age of 5 years, and they therefore provide stable reference points on which all subsequent linear measurements were based [19].

As in previous research [14], the VerT-Pterygomaxillary fissure (Ptm) line was constructed parallel to VertT passing through point Ptm. The following linear measurements were used to assess sagittal relationships: ANS-VertT-Ptm, A-VertT, Pr-VertT, Id-VertT, B-VertT, and Pg-VertT.

In addition to analysis as per Baccetti et al. [16], the horizontal position of the mesial cusp of the maxillary first molar (U6-VertT) and the perpendicular distance between the mesial cusp of that tooth and the palatal plane (U6-PP) were measured. The following lines and angles were also considered: SNA, SNB, ANB, SN-GoGn, SN-PP, PP-GoGn, and U1-PP, and a Wits appraisal was performed. In addition, the horizontal displacement of the maxillary first molar was evaluated (U6 mesialization), net from the skeletal displacement of the upper jaw.

### Statistical methods

Each outcome was analysed in each patient by means of descriptive analysis by time, mean and standard deviation (std), as well as plots. The analysis aimed to investigate which outcome significantly changed over time, and which time-points were significantly different in terms of mean outcome. Repeated-measures ANOVA through a linear mixed model framework was used to model the relationship between the outcome and time-point [20]; pairwise comparison of time-points was performed using a post-hoc analysis implemented by the R software package estimated marginal means ® Core Team 2021). Statistical significance was assessed using a type I error threshold of $\alpha = 0.05$ (5%).

### Bias

All measurements were carried out by the same operator (M.D.). Measurement error (repeatability) has been assessed using the Dalhberg Formula [21], for descriptively measure the error size, as well as a repeated measures t-test, to check bias absence.

### Study size

The given sample size under the repeated measures ANOVA framework yields a minimum detectable effect size equal to $f = 0.612$, which, according to Ferguson [22], is over the large effect threshold.

## Results

### Participants

Cephalometric measurements were recorded for each of the 27 patients at T0 (Pre-treatment: mean age 11 years, 4 months), T1 (post-facemask treatment: mean age 12 years, 1 month) and T2 (follow-up: 7 years, 10 months after T1, mean age 19 years, 10 months). The repeatability of measurements is good as no t-test p-value is below significance threshold. Demographic information, mean and SD, for patients at T0, T1 and T2 are summarized in Table 1. As long as the mandibular growth pattern was considered, 20 patients were initially normodivergent, 5 hyperdivergent and 2 hypodivergent. At the end, 16 patients were normodivergent, 3 hyperdivergent and 8 hypodivergent, emphasizing a tendency of mandibular anterior rotation.

**Table 1:** Mean and SD age, sex and facial pattern for patients at T0 (pre-treatment), T1 (post-facemask treatment) and T2 (long-term follow up)

	T0	T1	T2
Age	11 years, 4 months ± 1 years, 10 months (8 years, 8 months–18 years, 10 months)	12 years, 1 months ± 1 years, 9 months (9 years, 5 months–19 years, 5 months)	19 years, 10 months ± 1 years, 9 months (15 years, 8 months–24 years, 3 months)
Gender	15 females (55,6%) e 12 males (44,4%)		
Facial pattern	Hyperdivergent: 18.52% Normodivergent: 74.07% Hypodivergent: 7,41%	Hyperdivergent: 29.63% Normodivergent: 62.97% Hypodivergent: 7.41%	Hyperdivergent: 11.11% Normodivergent: 59.26% Hypodivergent: 29.63%

### Descriptive data

**Fig. 3:**
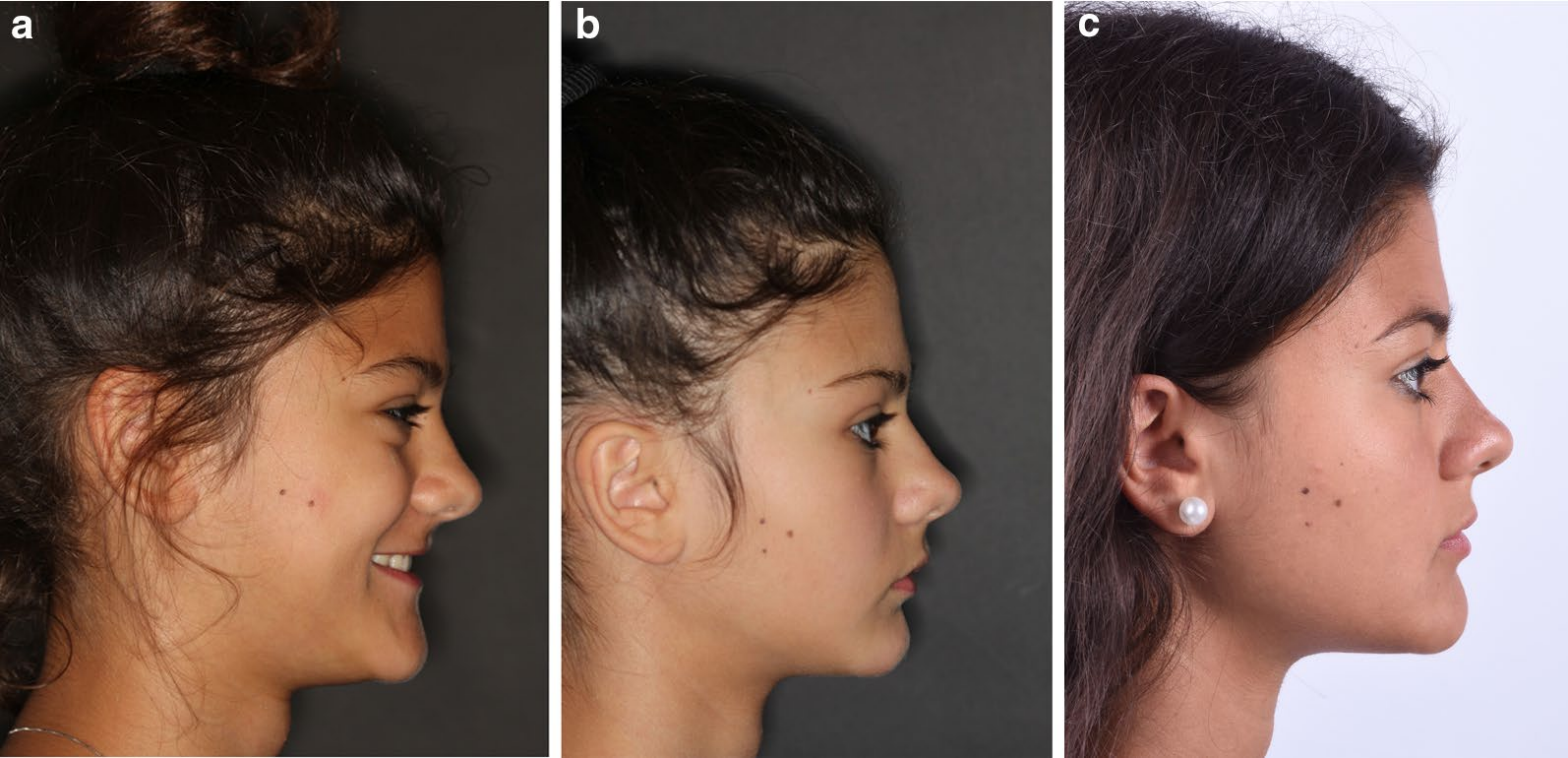
**a**–**c** Profile photographs of one patient of the study group at T0, T1, and T2

**Fig. 4:**
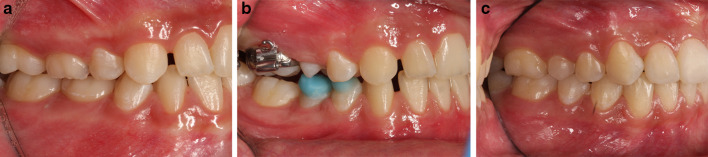
**a**–**c** Lateral occlusal photographs of one patient of the study group at T0, T1, and T2

**Fig. 5:**
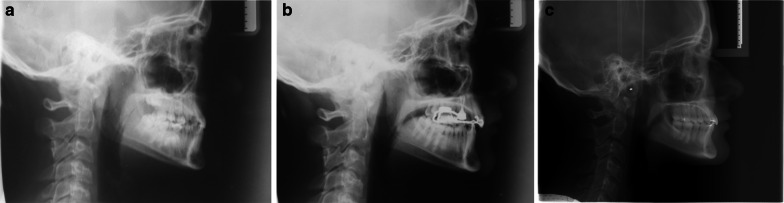
**a**–**c** Lateral X-rays of one patient of the study group at T0, T1, and T2

A descriptive statistical analysis was performed, considering Time as the variable; means and standard deviations for each outcome are reported in Table 2. A complete case is illustrated: profile photographs (Fig. 3a–c), lateral occlusal photographs (Fig. 4a–c) and lateral x rays (Fig. 5a–c) have been collected at T0 (14,1 years), T1 (15 years) and T2 (20,6 years).

**Table 2:** Descriptive analysis (mean and standard deviation) of the cephalometric measurements at T0 (pre-treatment), T1 (post-facemask treatment) and T2 (long-term follow up)

Outcome	T0	sd	T1	sd	T2	sd
A-VerT (mm)	53.22	5.1	56.73	6.1	55.99	4.5
B-VerT (mm)	50.23	6.2	50.50	7.4	49.45	8.0
ANS-Ptm (mm)	46.89	3.7	50.51	4.5	51.51	3.7
PNS-Ptm (mm)	2.36	1.1	3.47	1.7	3.90	1.2
Pr-VerT (mm)	54.73	5.8	58.72	6.7	56.54	5.8
Id-VerT (mm)	53.18	6.0	54.03	7.6	53.03	7.2
Pg-VerT (mm)	50.66	6.8	51.52	8.2	50.61	8.7
Wits appraisal (mm)	− 5.46	4.0	− 0.27	4.7	− 2.51	3.1
U6 vert PP (mm)	19.53	2.4	20.27	2.3	21.14	3.5
U6 mesialization (mm)	28.49	5.8	30.83	7.0	29.93	6.5
SNA (°)	79.72	3.8	82.76	3.3	82.25	3.6
SNB (°)	80.26	4.0	79.44	3.6	81.13	3.5
ANB (°)	− 0.56	2.4	3.31	2.3	1.12	2.0
PP-GoGn (°)	25.70	5.9	27.30	5.6	21.74	5.6
U1-PP (°)	111.9	6.1	109.7	6.8	115.4	7.2
SN-PP (°)	7.367	3.0	6.663	2.4	7.141	2.7
SN-GoGn (°)	33.09	5.6	33.99	5.5	28.90	5.6

*A* A-point; *B* B-point; *S* Sella; *N* Nasion; *ANS* anterior nasal spine; *PNS* posterior nasal spine; *Pr* Prosthion; *Id* Infradental; *Pg* pogonion; *PP* Palatal plane; *Go* Gonion; *Gn* Gnathion; *U6* upper first molar

### Main results

A post-hoc analysis was performed comparing the differences in means between the three possible pairs; the p-value indicates whether differences can be deemed statistically different from zero (Table 3). As the values show, after RME according to Liou’s protocol [23] and 4 months of facemask protraction, point A advanced by a mean of 3.5 mm with respect to VerT, then relapsed by 0.7 in the post-facemask period, thereby yielding of a mean advancement of 2.7 at T2. The variations from T0 to T1 and T0 to T2 are both significant, meaning that relapse from T1 to T2 was not statistically and clinically relevant. The differences between T1 and T2 depend from both the patient’s growth pattern but also from the biomechanics and camouflage of the orthodontic treatment.

**Table 3:** Pairwise post-hoc analysis comparing differences in time-point means

	T1–T0					T2–T0					T2–T1				
Outcome	Mean	SE	Lower CL	Upper CL	P level	Mean	SE	Lower CL	Upper CL	P level	Mean	SE	Lower CL	Upper CL	P level
A-VerT (mm)	3.516	0.6888	1.854	51.773	P < 0.05	2.773	0.6888	1.111	44.343	P < 0.05	− 0.743	0.6888	− 2.405	0.9188	NS
B-VerT (mm)	0.2726	1.264	− 2.777	3.322	NS	− 0.7815	1.264	− 3.831	2.268	NS	− 10.541	1.264	− 4.104	1.995	NS
ANS-Ptm (mm)	36.241	0.6418	20.756	5.173	P < 0.05	46.163	0.6418	30.678	6.165	P < 0.05	0.9922	0.6418	− 0.5562	2.541	NS
PNS-Ptm (mm)	11.122	0.3047	0.3770	1.847	P < 0.05	15.407	0.3047	0.8056	2.276	P < 0.05	0.4285	0.3047	− 0.3067	1.164	NS
Pr-VerT (mm)	3.989	0.9094	17.951	61.834	P < 0.05	1.806	0.9094	− 0.3886	39.997	NS	− 2.184	0.9094	− 43.778	0.0104	NS
Id-VerT (mm)	0.8433	1.165	− 1.967	3.654	NS	− 0.1526	1.165	− 2.963	2.658	NS	− 0.9959	1.165	− 3.806	1.814	NS
Pg-VerT (mm)	0.8637	1.382	− 2.470	4.197	NS	− 0.0470	1.382	− 3.381	3.287	NS	− 0.9107	1.382	− 4.244	2.423	NS
Wits appraisal (mm)	5.189	0.6893	3.526	68.519	P < 0.05	2.941	0.6893	1.278	46.037	P < 0.05	− 2.248	0.6893	− 3.911	− 3.261	P < 0.05
U6 vert PP (mm)	0.7352	0.5151	− 0.5074	1.978	NS	16.093	0.5151	0.3666	2.852	P < 0.05	0.8741	0.5151	− 0.3685	2.117	NS
U6 mesialization (mm)	23.456	1.36	− 0.9356	5.627	NS	14.415	1.36	− 18.397	4.723	NS	− 0.9041	1.36	− 41.852	2.377	NS
SNA (°)	30.333	0.4571	1.931	41.361	P < 0.05	25.259	0.4571	1.423	36.287	P < 0.05	− 0.5074	0.4571	− 1.610	0.5954	NS
SNB (°)	− 0.8148	0.372	− 17.123	0.0827	NS	0.8704	0.372	− 0.0272	17.679	NS	16.852	0.372	0.7877	25.827	P < 0.05
ANB (°)	3.870	0.3934	29.212	4.819	P < 0.05	1.681	0.3934	0.7323	2.631	P < 0.05	− 2.189	0.3934	− 31.380	− 1.240	P < 0.05
PP-GoGn (°)	1.593	0.7658	− 0.2551	3.440	NS	− 3.967	0.7658	− 58.143	− 2.119	P < 0.05	− 5.559	0.7658	− 74.069	− 3.712	P < 0.05
U1-PP (°)	− 2.170	1.339	− 53.996	1.059	NS	3.548	1.339	0.3189	6.777	P < 0.05	5.718	1.339	24.893	8.948	P < 0.05
SN-PP (°)	− 0.7037	0.4523	− 17.950	0.3876	NS	− 0.2259	0.4523	− 13.172	0.8654	NS	0.4778	0.4523	− 0.6135	15.691	NS
SN-GoGn (°)	0.8074	0.7146	− 0.9166	2.531	NS	− 42.889	0.7146	− 60.129	− 2.565	P < 0.05	− 50.963	0.7146	− 68.203	− 3.372	P < 0.05

*P* < 0.05: statistically significant; *NS* Not significant

95% of the contested time points difference and the modelled standard deviation (SE)

## Discussion

### Key results

In 2018, Maino et al. [14] reported positive short-term results in a group of patients treated with a hybrid-anchorage RME followed by 4 months of facemask treatment, until a hypercorrection was obtained (head-to-head molar relationship). The Alt-RAMEC/FM protocol’s efficiency is confirmed by the results obtained at T1 in the current sample study, with a mean point A advancement of 3.5 mm with respect to VerT. Moreover, all positive orthopaedic outcomes observed after Alt-RAMEC/FM treatment have been maintained until the end of mandibular growth.

### Interpretation

According to Baccetti et al. [16], significant forward displacement of maxillary structures can be achieved when tooth-borne maxillary expansion and facemask therapy are performed in early age [11]. Two different multicentre randomized controlled trial with respectively 3- and 6-year follow up confirmed the favourable effects of early class III protraction facemask treatment undertaken in patients under 10 years of age [24]. Even though a consistent relapse of skeletal cephalometric changes occurs, this early treatment reduces the need for orthognathic surgery in adult age [25]. On the other hand, late treatment yields no significant improvement in maxillary growth, just dentoalveolar changes with respect to controls.

Because the mean age was higher in this sample with respect to Baccetti et al.’s late treatment group [16], an Alt-RAMEC approach was applied, in which the anterior movement of point A was reported as being approximately twice (4.13 mm) that of the traditional expansion protocol (2.33 mm) [26]. The same approach was assessed in this longer-term retrospective study, in which 27 patients were re-evaluated at the end of mandibular growth, more than 7 years after the end of maxillary protraction. In the meantime, each patient underwent a standardized orthodontic treatment. Current cephalometric short-term results were similar to those reported in the meta-analysis conducted by Cordasco et al. [2] in terms of both sagittal and vertical measurements. However, the mean treatment duration in the present study was 4 months, as compared to 1 year in the articles cited by Cordasco et al. Moreover, the mean age of this sample was considerably greater (11 years vs. 8 years) than in the above-mentioned studies [2, 16]. Nonetheless, significant sagittal skeletal improvement was achieved after 4 months of protraction, as shown by changes in the SNA (3°) and Wits appraisal (5.2 mm). These results are more pronounced with respect to those reported by Nienkemper et al. [27] in 2015. In that case, despite the use of the same appliance (hybrid RME + facemask), the SNA increased by just 2.4 mm and the Wits appraisal by 4.5 mm. It is likely that this difference may be ascribable to the systematic application of the Liou protocol to activate the maxillary sutures before protraction in the current study [9, 10, 23].

Due to the residual mandibular growth in Class III patients, the treatment stability represents a great challenge often associated with a high rate of relapse. A recent systematic review with meta-analysis reported that the anteroposterior benefits related to RME + FM gradually relapse in the long-term follow-up period. Meta-regression analysis also showed that if the follow-up control takes place after more than 3 years, the effectiveness of maxillary protraction decreases even more [28]. The overall worsening of the cephalometric values, when a long follow-up is considered, depends on whether growth peak in Class III subjects is delayed and more pronounced compared to Class I subjects [1].

However, long-term results reported for this study sample at the end of growth (mean age: 19 years, 10 months) demonstrate the overall effectiveness and stability of the approach used. Although the long-term assessment highlights that a certain amount of relapse occurred, the ANB and Wits appraisal values were still significantly improved seven years after the end of the treatment, with the former displaying a mean increase of 1.7°, and the latter of 2.9 mm with respect to T0. According to Eslami et al. [29] Wits appraisal greater than—5.8 mm can be effectively treated by camouflage, whilst more negative values than—5.8 mm must be treated by surgery. At T2, just one patient of the sample reported the need for orthognathic surgery (Wits: − 14.80 mm). All the others have been successfully treated with orthodontic camouflage.

Moreover, the total advancement of point A at T2 was 3 mm, even considering the slight relapse recorded over time (0.7 mm in 7 years). A statistically significant growth of both Pg and B point in the long term was not reported, meaning that most of mandibular growth was already achieved during the orthopaedic treatment for the majority of patients. Meazzini et al. [30] have previously reported the short and long-term results for the application of the Alt-RAMEC technique in patients with skeletal Class III malocclusion, but in that case the maxillary protraction was performed by means of a pair of noncompliant tooth-borne springs. According to those authors, a 2-hinged expander enables better loosening of all circumaxillary sutures, thanks to its specific design [9, 31]. The application of two noncompliant tooth-borne springs lead to slightly more pronounced maxillary advancement with respect to the one achieved by our sample (5.43 mm versus 3.5 mm). This may be related to the fact that no collaboration was required in the treated group reported by Meazzini et al. [30]. On the other hand, the efficacy of facemask highly depends on the patient’s collaboration. The lack of molar mesialization is probably the reason why Meazzini et al. found 2.1° of lower incisors’ lingual inclination subsequent to the upper lip pressure on the upper incisors [32]. 4 mm of forward displacement of upper incisors was observed as well. Foersch et al. [11] reported labial inclination of maxillary incisors in patients treated via traditional facemask approach. That being said, in the current study the lingual inclination relapsed significantly in the follow-up, with an overall increase of 3.5 of the U1-PP value, proving that the orthodontic treatment resulted in a slight dental compensation of the Class III malocclusion. Finally, as far as the vertical dimension is concerned, clockwise rotation of the mandible (SN-GoGn: + 0.9°) and extrusion of the U6 (0.7 mm) was shown in this sample, which contributed to correction of the Class III sagittal relationship. This is in line with findings reported by other researchers using bone-anchored devices for maxillary protraction [33, 34]. At the end of the follow-up, counter-clockwise rotation of the mandible was also appreciable (SN-GoGn: − 5°), ascribable to entity and direction of the mandibular residual growth, perfectly in line with the other recent long-term findings by Meazzini et al. (Sn-GoGn: − 2.14°) [28].

### Generalisability

Positive outcomes correlated to the application of tooth-borne rapid palatal expander and different Class III appliances are shown in all these studies mentioned [10, 16, 28]. However, some important variables (for example, initial mean age, type of appliances, grade of collaboration) influence the final results, and make the comparison more difficult. More recently, Papadopoulou et al. [35] analysed the long-term effects of a different approach, characterized by hybrid-Hyrax, alt-RAMEC and intraoral Class III elastics anchored to miniscrews-reinforced-Lower-lingual-Arch (alt-RAMEC, HH-LLA), followed by fixed multibrackets appliance. At baseline, the mean age of the 15 patients analysed was similar to the one of the present study. The post-pubertal skeletal and overjet corrections were consistent, but the sagittal skeletal improvement less pronounced, with a minor increase of ANB angle and Wits appraisal. Because no intermediate cephalometric tracings were collected, the long-term results are not attributable to the first orthopaedic or the second orthodontic phase, but a combination of both.

### Limitations

Being a retrospective clinical study, it has some limitations and risk of bias, in particular correlated to the absence of a control group and randomization protocol. As Table 2 shows, the majority of mean values have a high standard deviation, emphasizing a significant inter-individual variability. This may explain why no statistically significant difference in point B and Pg position was reported at the end of follow up period, even though some singular patient did show a residual mandibular growth. Moreover, the final results highly depend from the orthodontic biomechanics as well. Despite all limits, the above results may be of clinical interest, especially considering the short-term duration of orthopaedic treatment, the initial high mean age, and the long follow-up until the end of growth.

## Conclusions

Combining hybrid expansion and Alt-RAMEC protocol followed by facemask protraction corrected the skeletal Class III through consistent maxillary advancement. The short-term results are related to the only Skar III + FM approach, while the long-term are both related to the combined effects of the orthopaedic correction, growth and fixed orthodontic treatment. Despite a slight relapse, the long-term cephalometric follow-up confirms the maintenance of all positive outcomes of the previous orthopaedic treatment until the end of mandibular growth.

## Data Availability

The dataset used and/or analysed during the current study is available from the corresponding author on reasonable request.
